# Unique Organic–Inorganic Hybrid Copper(I) Phosphate with Ultralow Ractopamine Detection Limit and In Situ Sensing Ability

**DOI:** 10.1021/acs.inorgchem.4c05123

**Published:** 2025-02-27

**Authors:** Ji-Fang Xie, Pi-Chen Wei, Ying Li, Chiao-Chun Chang, Kai-Chi Chang, Ching-Ping Lu, Todd Hsu, Der-Lii M. Tzou, Hsiung-Lin Tu, Chih-Min Wang

**Affiliations:** † Department of Bioscience and Biotechnology, National Taiwan Ocean University, Keelung, Taiwan 202, ROC; ‡ Department of Applied Chemistry, National Chi Nan University, Nantou, Taiwan 202, ROC; § Department of Environmental Biology and Fisheries Science, National Taiwan Ocean University, Keelung, Taiwan 202, ROC; ∥ Institute of Chemistry, Academia Sinica, Taipei, Taiwan 202, ROC; ⊥ General Education Center, National Taiwan Ocean University, Keelung, Taiwan 202, ROC

## Abstract

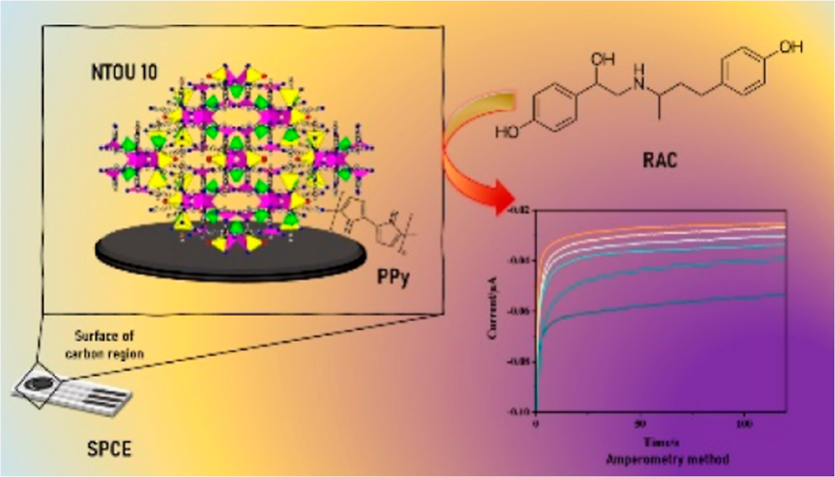

We successfully synthesized and characterized the first-ever organic–inorganic hybrid metal phosphate, **NTOU-10**, featuring lower-valence copper nodes under hydrothermal conditions. The crystalline structure, confirmed through single-crystal X-ray diffraction, thermogravimetric analysis, solid-state NMR spectroscopy, X-ray photoelectron spectroscopy, and bond valence-sum calculations, revealed its chemical composition, and unique coordination bonding models between organic linkers, metal nodes, and phosphate groups. These building blocks intricately bonded through metal–oxygen and metal–nitrogen linkages to form a highly complex crystalline structure. Taking innovation a step further, we polymerized **NTOU-10** with pyrrole to develop the hybrid **NTOU-10@polypyrrole** (PPy) electrode, setting a groundbreaking record with an ultrasensitive limit of detection (LOD) of 3.31 × 10^–19^ M for ractopamine (RAC) sensing, surpassing existing crystalline materials by more than 9 orders of magnitude. The real-world effectiveness of this biosensor was demonstrated by detecting RAC in spiked pork samples. Additionally, we integrated the **NTOU-10@PPy** electrode into a flow injection analysis (FIA) system, offering advantages such as rapid detection, minimal sample requirement, and operational convenience. This work presents a significant leap forward in the design and synthesis of novel materials, along with the development of a high-performance biosensing platform for RAC detection.

## Abstract 2

The first-ever crystalline organic−inorganic hybrid metal phosphate incorporating lower-valence copper atoms as metal nodes has been successfully synthesized under hydrothermal conditions. This remarkable compound showcases a versatile range of coordination types between organic linkers, metal nodes, and phosphate groups within its crystalline structure. In a parallel breakthrough, we developed a hybrid **NTOU-10@polypyrrole** electrode, leading to the creation of a cutting-edge biosensor platform. This platform set a new benchmark, achieving an unprecedented detection limit of 3.31 × 10^−19^ M for ractopamine, outshining any crystalline material by over 9 orders of magnitude. With this innovation, we’ve not only crafted a material with a unique structure and composition but also opened new, efficient pathways for detection methods that offer unparalleled convenience, speed, minimal analyte requirements, and exceptional signal clarity and resolution.

## Introduction

In the dynamic field of chemical sensing and detection, the relentless pursuit of cutting-edge innovations has given rise to the development of novel materials with remarkable properties.^1,2^ Among these, crystalline compounds such as coordination polymers, metal–organic frameworks, hybrid metal phosphates, and phosphites are leading the charge. These materials exhibit extraordinary structural features, opening doors to a wide range of applications, including adsorption, luminescence, detection, pollutant removal, and energy technologies.^1−16^ As the demand for high precision and sensitivity in chemical analysis intensifies, the need for groundbreaking sensing platforms becomes paramount. This paper reports the synthesis, and characteristics of a new crystalline material, as well as its groundbreaking capabilities of an innovative organic–inorganic hybrid copper(I) phosphate, emphasizing the potential to revolutionize the landscape of detection abilities, not only in terms of detection limits but also for the in situ sensing properties. The amalgamation of organic and inorganic components imparts unique structural features and characteristics to this material, setting it apart from other sensor matrices. Understanding and harnessing those new structures are also essential for exploring and unraveling the material’s exceptional sensing capabilities. For example, we recently reported an indium phosphate (NTOU-7) with a very good detection limit of 2.74 × 10^–10^ mol/L for ractopamine.^17^ This drug, being a potent β-adrenergic agonist, necessitates highly sensitive detection methods due to its regulatory importance in the food and pharmaceutical industries.^18,19^ The NTOU-7-based microfluidic biosensor also demonstrated excellent property in detecting dopamine.^20^ In this report, the innovative **NTOU-10@PPy** hybrid electrode was developed by seamlessly integrating **NTOU-10** with pyrrole through polymerization. This advanced hybrid electrode was then combined with a screen-printed carbon electrode (SPCE), creating a highly efficient and responsive sensor poised to redefine electrochemical detection performance. Significantly, this hybrid biosensor containing **NTOU-10** distinguishes itself through its revolutionary ultralow detection limit (3.31 × 10^–19^ M), surpassing all known crystalline compounds by more than 9 orders in the detection of ractopamine. This exceptional capability represents a crucial attribute in the identification of this biologically active compound. We also incorporated this finding into a flow injection analysis (FIA) system to develop a new platform that offers several advantages, such as rapid detection, minimal sample requirements, and ease of operation, comparable with the traditional precision methods like high-performance liquid chromatography (HPLC), liquid chromatography–mass spectrometry (LC–MS), gas chromatography–mass spectrometry (GC–MS), and enzyme-linked immunosorbent assay (ELISA) for the analysis of veterinary drug residues.^21−24^ To the best of our understanding, this case shows a very rare instance of an organic–inorganic hybrid metallophosphate that integrates copper(I) ions as metal nodes, phosphates, and 4,4′-(1*H*-1,2,4-triazole-3,5-diyl)dipyridine (HTDP) building units, showcasing an unparalleled structure, the ultralow limit of detection, and remarkable in situ sensing capabilities. Traditionally, Cu^2+^ ions have been regarded as more stable copper cations than Cu^1+^ due to their electronic configuration and hydration energy. The occurrence of crystalline materials containing copper(I) is also noted to be less frequent when compared to those incorporating copper(II).^3,7^ Herein, we reported the synthesis, structural features, and properties of a novel copper(I) phosphate (denoted as **NTOU-10**). Furthermore, the fabrication process of the hybrid device utilizing this compound for electrochemical detection and in situ sensing studies, was also elucidated.

## Experimental Section

### Synthesis and Initial Characterization

**Figure 1:**
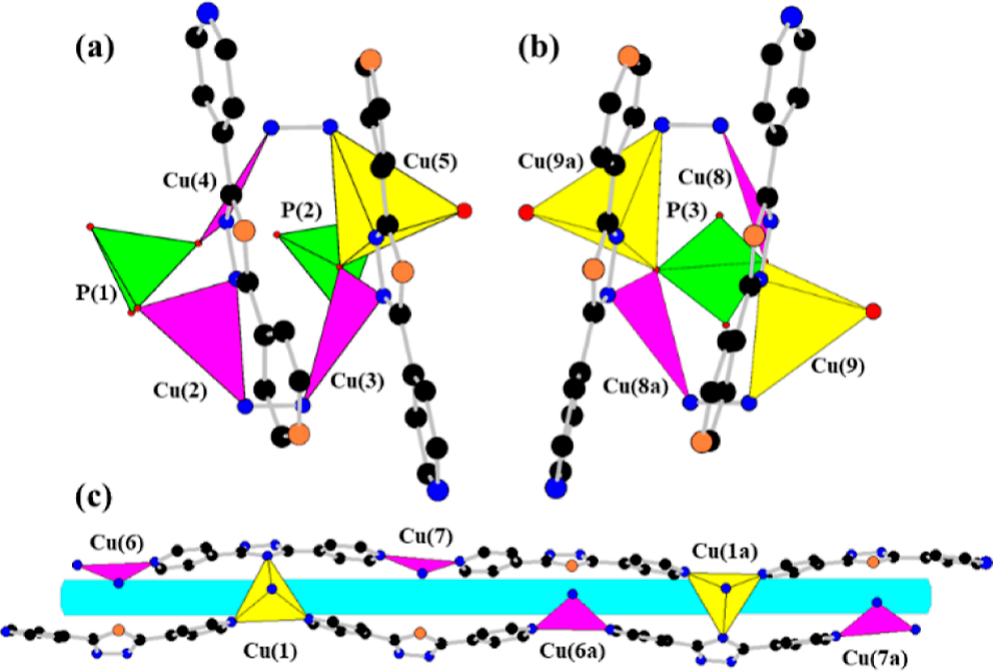
Coordination environment for copper and phosphorus atoms, as well as the organic ligands in **NTOU-10**, is depicted with color-coded atoms for clarity. The different subunits of **NTOU-10** are presented in panels (a), (b), and (c), respectively. Blue, black, and red circles represent nitrogen (N), carbon (C), and oxygen (O) atoms, respectively. The coordinated water oxygen atoms and nitrogen atoms of organic molecules not bonding to metals are separately in large red and orange circles. Phosphorus and copper polyhedra are depicted in green and yellow (or pink), respectively. Hydrogen atoms are omitted for visual simplicity.

Red-black crystals of **NTOU-10** were synthesized in Teflon-lined stainless-steel Parr acid digestion bombs (23 mL autoclaves) by heating a mixture of Cu(NO_3_)_2_·3H_2_O (0.25 mmol), H_3_PO_4_ (0.25 mmol), HF (aq) (1 mmol, 48% solution), HTDP (0.25 mmol), H_2_O (1 mL), and 1,4-butanediol (3 mL) at 150 °C for 2 days. To obtain high-quality crystals suitable for single-crystal X-ray diffraction (SXRD) analysis, the cooling rate was carefully controlled at 2 °C/h until the temperature dropped to 30 °C. The crystalline product was filtered, washed with water and ethanol, and dried in an oven. Powder X-ray diffraction (PXRD) data were collected using a Bruker D2 Phaser X-ray diffractometer with Cu Kα radiation. The PXRD pattern of the bulk product matched the calculated pattern derived from single-crystal X-ray diffraction data for **NTOU-10** (Figure S1). Elemental analysis results were consistent with the expected formula [anal. found (calcd): C, 39.29% (38.97%); H, 2.63% (2.75%); N, 19.09% (18.93%)], and the yield was 83% based on Cu. Thermogravimetric analysis (TGA) was performed on a powder sample of **NTOU-10** using a PerkinElmer TGA7 thermal analyzer under flowing oxygen at a heating rate of 5 °C/min from 40 to 900 °C (Figure S2). The first weight loss of 3.24% between 40 and 240 °C confirmed the presence of two lattice and two coordinated water molecules per formula unit (3.24%). The X-ray powder pattern of the final decomposition product from the TG analysis indicated the presence of CuO (JCPDS: 05-0661) and Cu_5_P_5_O_10_ (JCPDS: 31-0472). The calculated weight loss of 62.45% was slightly different from the observed value (59.19%) between 40 and 900 °C, likely due to the oxidation of cuprous species, dehydration of phosphate groups, and formation of copper oxides and phosphates during decomposition (2.34% + 59.19% = 61.53%). According to the TGA and PXRD analyses (Figures S2 and S3), **NTOU-10** maintained its structural framework up to 350 °C and exhibited water stability under continuous stirring for 14 days. A solid-state NMR study was performed using a Bruker Advance 300 MHz spectrometer (Figure S4). The ^31^P cross-polarization (CP) MAS NMR spectrum was recorded with ^1^H and ^31^P Larmor frequencies of 300.13 and 121.49 MHz, respectively. A 90° pulse with an rf field strength of 59.5 kHz was applied to the ^1^H channel, with a pulse sequence delay of 4 s. The ^1^H/^31^P polarization transfer was optimized to satisfy the Hartman-Hahn matching condition. A contact time of 1 ms was used for the polarization transfer, with rf field strengths of 46.2 kHz applied to both the ^1^H and ^31^P channels. During data acquisition, two-pulse phase-modulated (TPPM) ^1^H decoupling was employed with an rf field strength of 76.9 kHz. The powdered sample was packed in double-bearing 4 mm zirconium oxide MAS rotor and the MAS sample spinning rate was set to 10.0 kHz, regulated by a spinning controller to within ±1 Hz. The ^31^P chemical shifts were references to the ^31^P signal of Na_2_HPO_4_ at 5.5 ppm.^17^ Previously, the NMR spectrum of a gallium phosphate with four ^31^P peaks at −0.4, −9.2, −11.3, and −17.9 ppm indicated the existence of the different phosphate groups of H_2_PO_4_, HPO_4_, H_0.5_PO_4_, and PO_4_ in the crystalline structure, respectively.^25^ The isotropic chemical shift of ^31^P moves toward the higher field value with decreasing protonation. Recently, we reported an indium phosphate with the structural building units of H_1.5_PO_4_ based on bond valence-sum calculation, NMR, and SXRD analyses.^17^ In this case, the cross-polarization magic-angle-spinning ^1^H/^31^P NMR spectrum displayed main signals at about 11.0, 7.0, and 1.5 ppm with integrated intensities of about 1:2:2 (Figure S4), which were agree with the SXRD results for three kinds of phosphorus coordination environments in **NTOU-10**. X-ray photoelectron spectrum (XPS) was used to confirm the oxidation state of copper metal in the composition of **NTOU-10**. The observation (Figure S5) of two main peaks at 931.5 and 951.3 eV is closed to the previous study of Auger parameter values of 2p_3/2_ and 2p_1/2_ for the characteristic of Cu^1+^.^26^ The bond valence calculation also confirmed the copper atoms of **NTOU-10** existed in the lower oxidation state +1 (the values varied between 0.75 and 0.86 for copper metals).^27^ For single-crystal X-ray crystallography, a red-brown crystal of dimensions 0.15 × 0.03 × 0.02 mm^3^ was selected for indexing and intensity data collection on a Rigaku XtaLAB Synergy DW diffractometer. The number (15,054) of unique reflections [(*F*_o_) > 4σ(*F*_o_)] was collected with θ_max_ = 77.19° and *R*_int_ = 0.0380.^28^ The SADABS program was applied for the absorption correction. The values of *T*_min_ and *T*_max_ are 0.866 and 0.923, respectively. In the light of systematic absences, successful solution, and refinement of the crystalline structure, the space group was determined to be *I*2/*a* (no. 15). The structure of **NTOU-10** was solved using the direct method, with all atoms except the P(3) atom located in general positions. The metal and phosphorus atoms were identified first, followed by the O, C, and N atoms, which were located from difference Fourier maps. The N(2), N(6), N(7), N(12), N(15), N(22), N(26), and N(27) atoms of the TDP ligands were treated as terminal ends, while the other nitrogen atoms were bonded to copper atoms in the structure. The hydrogen atoms bonded to the carbon atoms of the TDP molecules were positioned geometrically and refined using a riding model. As shown in Figure 1a,b, each phosphorus atom adopts a tetrahedral geometry, bonding to two copper atoms through oxygen atoms, leaving terminal P–O groups [P(1) to Cu(2) and Cu(4); P(2) to Cu(3) and Cu(5); P(3) to Cu(8) and Cu(9)]. The bond lengths for the terminal P–O groups range from 1.526 to 1.596 Å. The longer bond lengths of P(1)–O(4) and P(2)–O(6) are 1.596 and 1.578 Å, respectively, and the hydrogen atoms associated with both oxygen atoms were located in difference Fourier maps. Determining the bonding types for the other terminal oxygen atoms, P(1)–O(2), P(2)–O(7), P(2)–O(8), and P(3)–O(10), with bond lengths of 1.533, 1.556, 1.526, and 1.548 Å, is ambiguous due to the scrambling/disorder of P–O–/P=O characteristics.^29^ Bond-valence calculation showed the four atoms with the unsaturated values of 1.22, 1.14, 1.24, and 1.16 for O(2), O(7), O(8), and O(9), respectively. Therefore, except for O(2), and O(8), they are assigned to be the OH/OH_0.5_ groups. Additionally, on the basis of the results for the charge-balance requirement, bond valence-sum calculation, NMR, XPS, EA, TGA, and SCXD analyses, the chemical formula of **NTOU-10** can be determined as Cu_9_(H_2_O)_2_(HTDP)(TDP)_5_(H_2_PO_4_)_0.5_(H_1.5_PO_4_)(HPO_4_)·2H_2_O. The final cycles of least-squares refinement with the atomic coordinates and anisotropic thermal parameters of all non-hydrogen atoms converged at *R*_1_ = 0.0414, w*R*_2_ = 0.1097, and *S* = 1.0227. All calculations were performed using the PC version of the Olex2 program package. The crystal data of **NTOU-10** (CCDC-2383542) can be obtained by emailing data_request@ccdc.cam.ac.uk, or by contacting The Cambridge Crystallographic Data Centre, 12 Union Road, Cambridge CB2 1EZ, UK; fax: +44 1223 336033. The crystallographic data, selected bond lengths and bond angles are given in Tables S1–S3, respectively.

## Results and Discussion

### Structure

**Figure 2:**
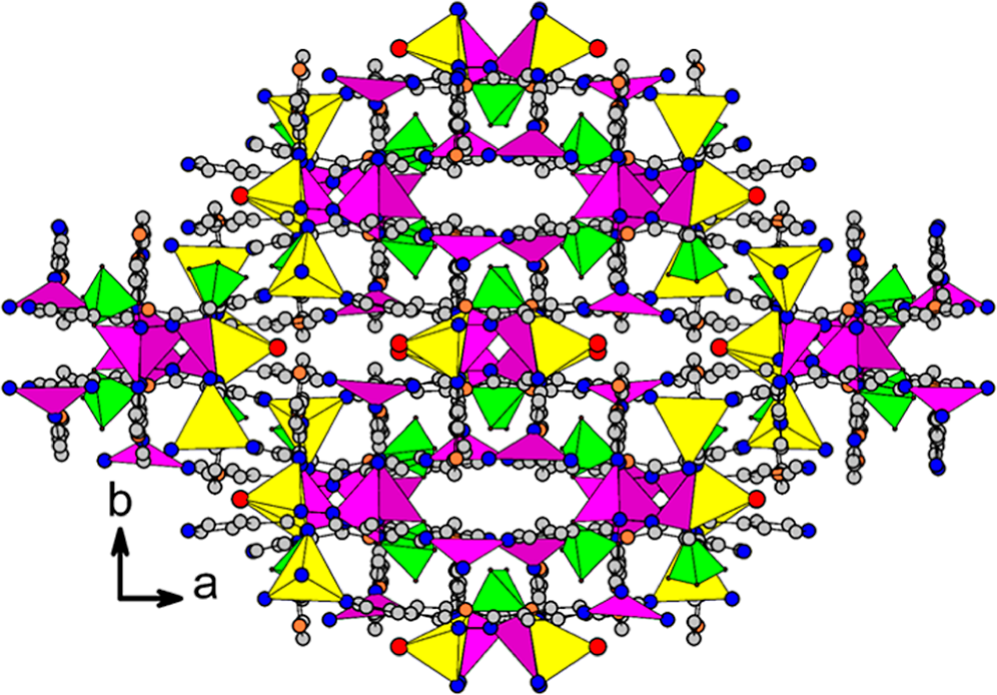
A complex structure of **NTOU-10** viewed along the *c*-axis, with the carbon atoms of the organic ligands represented by gray circles. For clarity, disordered lattice water molecules and hydrogen atoms have been omitted.

As illustrated in Figure 1, the **NTOU-10** framework is composed of nine copper atoms, three phosphate groups, and six organic ligands. The Cu(1), Cu(5), and Cu(9) atoms exhibit 4-coordinate geometries, forming CuN_4_ or CuN_2_O(H_2_O) tetrahedra. Cu(1) is coordinated by nitrogen atoms from the organic linkers, while Cu(5) and Cu(9) are bonded to a phosphate oxygen, one water molecule, and two nitrogen atoms from TDP. The other copper atoms adopt a trigonal planar coordination geometry, with nitrogen (from organic ligands) and/or oxygen (from phosphate units) atoms forming the vertices. Phosphate tetrahedra P(1) and P(2) each share two bridging and one μ_2_-oxygen atoms with copper polyhedra, creating distinct connection types of inorganic metallophosphate trimers (Figure 1a). Phosphate P(3) is bonded to four copper atoms via two μ_2_-oxygen atoms, generating another structure-forming pentamer comprising one phosphate and four copper polyhedra (Figure 1b). The terminal oxygen atoms of the phosphates are either double-bonded oxygens or hydroxyl groups within the structure. Notably, the multidentate 3,5-dipyridyl-1,2,4-triazole molecules exhibit versatile coordination modes as tri-, tetra-, and pentadentate ligands, linking the copper atoms to form the structural subunits of **NTOU-10**, as seen in Figure 1. The infinite chains (Figure 1c) play a crucial role in connecting the two building units (Figure 1a,b), resulting in a complex organic–inorganic hybrid framework that contains two lattice water molecules within its voids (Figures 2 and S6). Few crystalline materials consisting of copper atoms and phosphate groups have been reported.^30−42^ Among these, crystals with an oxidation state of +2 are common. Research shows that only three zeolites with a copper(II) phosphate composition are listed in the database of zeolite structures, with framework type codes ABW, AFI, and ATO.^43^ Inorganic compounds of lithium copper(I) phosphates with three different composition ratios of Li_3–*x*_Cu_*x*_PO_4_, along with Cu_3_(PO_4_) as a metastable product, were obtained under equilibrium investigations for the quasi-binary system of lithium phosphate and copper phosphate.^41^ Another mixed-valent copper orthophosphate, [Na_2_Cu^II^_3_(PO_4_)_2_][Cu^I^OCl], has also been discovered and structurally characterized.^31^ In comparison, crystalline materials containing copper atoms as coordination nodes in the construction of organic–inorganic hybrid metallophosphate and/or metallophosphite structures are limited.^3,7,30−42^ The first example of an organically templated copper(II) phosphate featured an infinite chain of edge-sharing [CuO_4_Cl_2_] polyhedra, decorated by one- and two-connected H_2_PO_4_ tetrahedra.^35^ A few examples of mixed-metal phosphates with multidimensional structures formed by the linkage of organic ligands, copper(II) and other metal atoms have been reported.^32−34,36,37,40,42^ For instance, vanadium phosphate units have been bonded through copper(II) coordination complexes such as {Cu(4,4′-bipy)}^∞^ chains, {Cu(1,10-phen)^2+^}, and {Cu(2,2′-bipy)^2+^} fragments to generate three mixed-metal compounds, one of which exhibited antiferromagnetic properties due to 1D magnetic interactions between dinuclear copper units.^33,37,42^ Additionally, three isostructural compounds with the mixed-metal compositions M(VO_2_)_2_(4,4′-bipy)_2_(HPO_4_)_2_ (M = Co, Ni, and Cu) feature 2D layers of metal phosphate linked by neutral 4,4′-bipy pillars.^32^ Our previous reports also described two hybrid phosphates comprising bimetallic oxide layers coordinated by isonicotinate and 4,4′-bipyridine ligands, forming 2D and 3D structures, respectively.^34,36^ One structural unit of uranium phosphate was bonded with copper(II) coordination {Cu(4,4′-bipy)}^∞^ chains, resulting in a pillared layer framework.^40^ To our knowledge, no hybrid phosphates or phosphites containing cuprous atoms as nodes to extend their structural dimensions into organic–inorganic frameworks have been found. This may be due to the tendency of Cu(I) to oxidize to Cu(II) under various conditions, making the crystallization of such materials with cuprous atoms challenging. Precise control in synthesis is crucial, as even slight variations in the quantities of reactants (such as phosphate and ligand), solvent, and pH value can significantly affect product purity, often resulting in unknown substances. This unusual example of the titled compound features copper in a lower oxidation state and includes diverse coordination types of ligands, metal nodes, and phosphate groups in its crystalline structure, which was observed for the first time.

### Detection

**Figure 3:**
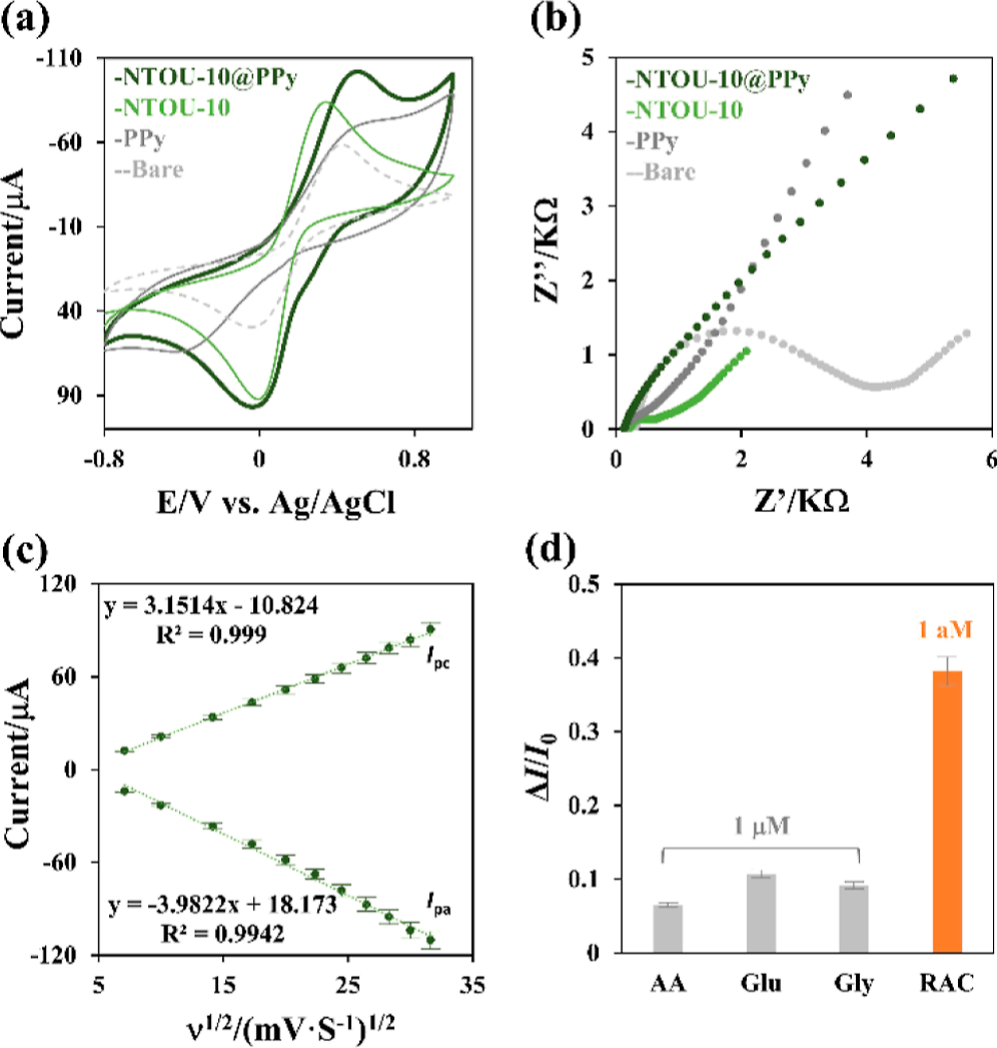
Electrochemical properties of the **NTOU-10@PPy** hybrid electrode: (a) CV spectra of the bare electrode, PPy, **NTOU-10**, and **NTOU-10@PPy** recorded over a potential range of −0.8 to 1.0 V at a scan rate of 0.1 V s^–1^ in pH 7.0 PBS. (b) Nyquist plots of the bare SPCE and modified electrodes (**NTOU-10**, PPy, and **NTOU-10@PPy**) in 0.1 M KCl solution containing 5 mM Fe(CN)_6_^3–/4–^. (c) Calibration curves showing the relationship between peak currents (Ipa and Ipc) and different scan rates. (d) Selectivity of the **NTOU-10@PPy** for RAC detection, tested in the presence of ascorbic acid (AA), glucose (Glu), and glycine (Gly).

**Figure 4:**
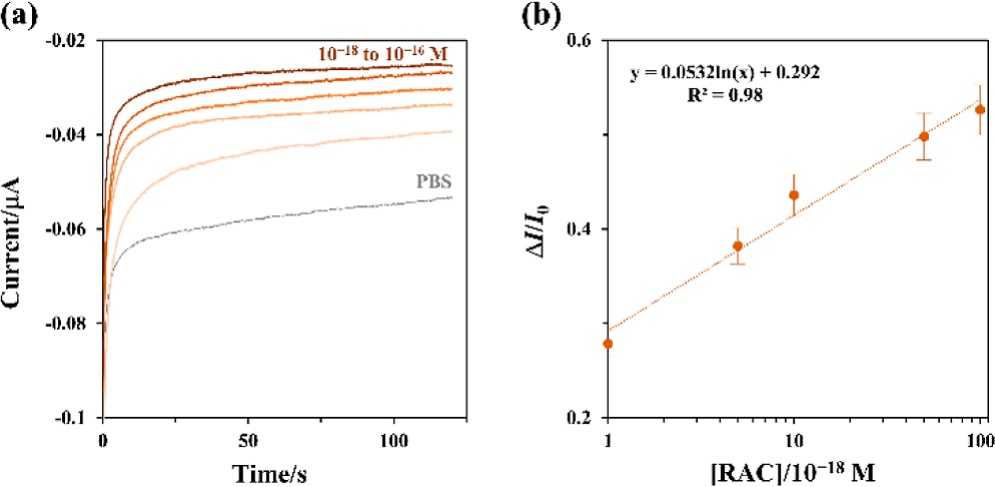
Amperometric method for RAC biosensor: (a) current responses recorded at 0.67 V in the presence of RAC at concentrations ranging from 10^–18^ to 10^–16^ mol L^–1^ (indicated by a gradient from light to dark color). (b) The calibration curve demonstrates a linear increase in current with increasing RAC concentrations. Three tests are for each concentration. The standard deviation (and standard error) values for each concentration, from low to high, are 4.7% (5.7%), 6.7% (8.2%), 4.3% (5.3%), 4.3% (5.3%), and 4.4% (5.4%), respectively.

The Codex Alimentarius Commission has set guidelines for detecting ractopamine (RAC), a feed additive used to promote leanness in meat-producing animals. The ability to detect ractopamine is crucial for ensuring food safety and protecting consumers, as residues of this substance in meat products may pose health risks.^18,19^ Therefore, the design and fabrication of novel materials and devices are critical not only for preventing illegal use of substances but also for maintaining public trust in food quality. To develop new platforms that overcome current detection limits, hybrid electrodes incorporating **NTOU-10** were prepared on either screen-printed carbon electrodes (SPCE) or custom multielectrode arrays using optimal protocols in a 0.1 M phosphate-buffered (PB) solution at pH 7.0 (see Supporting Information for detailed experimental procedures). Three potential components, polyglycolic acid (PGA), gold nanoparticles (Au NPs), and polypyrrole (PPy), were combined into the hybrid electrodes using layer-by-layer (LBL) assembly or copolymerization/deposition methods for detection studies (Figure S7). Differential pulse voltammetry (DPV) results demonstrated that **NTOU-10@PPy** exhibited higher sensitivity for detecting RAC at a concentration of 0.1 μM (Figures S8and S9), making it the material of choice for further studies. A fine powder of **NTOU-10** and pyrrole was coelectrochemically polymerized to form a hybrid **NTOU-10@PPy** nanocomposite using cyclic voltammetry (CV) under optimal conditions, with 1.5 mg mL^–1^ of **NTOU-10** and 0.1 M of pyrrole at 5 scanning cycles, and a scan rate of 100 mV s^–1^ within a potential range of −0.25 to 0.75 V (Figure S10). Dynamic light scattering (DLS) and scanning electron microscopy (SEM) showed that the particle size of **NTOU-10** was approximately 170 nm (Figures S11 and S12). The amount of **NTOU-10@PPy** deposited on the electrode surface during polymerization was calculated to be 2460 ng cm^–2^ using an electrochemical quartz crystal microbalance (EQCM) (Figure S13). The continuous polymerization of **NTOU-10** and pyrrole on the electrode surface likely leads to the formation of a core–shell hybrid nanocomposite via interfacial in situ synthesis (Figures S7c and S12). The **NTOU-10@PPy** film served as the working electrode, and its electrochemical properties were investigated using a conventional three-electrode system. CV curves (Figure 3a) for the bare, PPy, **NTOU-10**, and **NTOU-10@PPy** electrodes were analyzed in a 0.1 M PB solution (pH 7) containing 5 mM ferri-ferrocyanide [Fe(CN)_6_^3–/4–^]. The results showed typical redox activity with peaks in both positive and negative scans. The higher faradaic response of **NTOU-10@PPy** could be attributed to enhanced electron transfer at the **NTOU-10@PPy** interface, which was consistent with electrochemical impedance spectroscopy (Figure 3b). The linear relationship between peak currents (*I*_pa_ and *I*_pc_) and the square root of the scan rate (ν^1/^^2^) indicated that the electron transfer reaction of **NTOU-10@PPy** followed a diffusion-controlled quasi-reversible process (Figure 3c). Preliminary studies showed the superior sensing ability of **NTOU-10@PPy** for RAC, even in the presence of common interference like ascorbic acid (AA), glucose (Glu), and glycine (Gly) as determined by DPV (Figures 3d and S9). Additionally, amperometric i-t analysis was performed to achieve more accurate and practical measurements of RAC using the **NTOU-10@PPy** hybrid electrode. Current responses at different RAC concentrations, along with a calibration curve showing a linear range from 10^–18^ to 10^–16^ mol L^–1^, were presented in Figure 4. The ultralow limit of detection (LOD) of 3.31 × 10^–19^ M surpasses the performance of previously reported RAC detection methods (Table S4). To obtain more detailed information on the sensing relationship between RAC molecules and **NTOU-10** (or/and **NTOU-10@PPy**), Fourier transform infrared spectroscopy (FTIR) and single-crystal X-ray diffraction experiments were conducted. As shown in Figure S14, the FTIR spectra exhibited characteristic bands corresponding to RAC, PPy, and **NTOU-10**. For RAC, peaks at approximately 1516 and 1610 cm^–1^ were attributed to N–H stretching and C=C vibrations, respectively.^44^ In the PPy spectrum, signals around 1084 and 1660 cm^–1^ likely originated from C–H in-plane bending, ring deformation, and C=C/C–C in-ring stretching modes.^45^ The characteristic bands of **NTOU-10**, observed at 959 and 1063 cm^–1^, were associated with asymmetric P–O stretching vibrations.^46^ The signals at 1430 and 1446 cm^–1^ should be corresponded to C–C/C=C and C–N/C=N ring stretching in the skeletal structure.^44,45^ In the spectra of **NTOU-10@PPy** and **NTOU-10@PPy-RAC**, the retention of characteristic bands confirmed the successful synthesis of the hybrid electrode and the effective incorporation of RAC into **NTOU-10@PPy**. The shifts in the characteristic bands suggested the presence of complex interactions that might play a key role in triggering the detection reaction between **NTOU-10@PPy** electrodes and RAC molecules. Attempts to study the RAC adsorption sites of **NTOU-10** using SXRD were unsuccessful due to the poor quality of **NTOU-10**, which became unsuitable for SXRD analysis after being soaked in an aqueous solution containing RAC molecules. This electrode also demonstrated excellent performance in real pork samples, with an average recovery rate of 101.31% for detecting spiked RAC concentrations ranging from 1 to 100 pM (Table S5). To evaluate its convenience and practicality, the **NTOU-10@PPy** hybrid was integrated with multielectrode arrays into an electrochemical flow injection analysis (FIA) system. The microfluidic device had a regular shape and was fabricated from polydimethylsiloxane using a soft lithography method.^20^ It featured hybrid electrodes and one or more sets of three inlets and one outlet as microchannels. This platform was used to examine the **NTOU-10@PPy** biosensor’s ability to achieve real-time detection of RAC. The new design not only allowed for high temporal resolution (<5 s) of RAC signals but also enabled rapid detection (Figure S15).

## Conclusions

In summary, we successfully synthesized a novel organic–inorganic hybrid metal phosphate containing copper atoms in the low-valent oxidation state through a carefully controlled hydrothermal method. This breakthrough structure, formed using Cu(NO_3_)_2_·3H_2_O, H_3_PO_4_, HF, TDP, water, and 1,4-butanediol, represents one of the few examples of crystalline metal phosphates featuring lower-valence cuprous metals as nodes, expanding its framework dimensions. The compound also revealed a rich and intricate coordination chemistry among ligands, metal nodes, and phosphate groups, resulting in a complex architecture that is unprecedented in metallophosphate and metallophosphite systems. Simultaneously, we developed a cutting-edge **NTOU-10@PPy** hybrid electrode and biosensor that shattered detection limits, achieving a record-breaking LOD of 3.31 × 10^–19^ M for RAC detection. The innovative platform also offers significant advantages in terms of rapid detection, minimal analyte use, and convenience, paving the way for more precise and efficient sensing technologies. The design and synthesis of such groundbreaking materials not only push the boundaries of current scientific understanding but also hold immense potential for future applications. Ongoing efforts continue to explore new crystalline materials and advanced technologies to meet the evolving demands of detection and material science.

## Supporting Information Available

Experimental details for the synthesis of **NTOU-10**, preparation of hybrid electrode and platform, structures, solid-state NMR spectroscopy, electrochemical analysis, ractopamine detection, structural stability, TGA, PXRD, XPS, SEM, DLS, EQCM, and IR measurements are described in the Supporting Information (PDF)
