# Supplementary material for: Unique Organic–Inorganic Hybrid Copper(I) Phosphate with Ultralow Ractopamine Detection Limit and In Situ Sensing Ability
Source: Inorg Chem. 2025 Feb 27;64(9):4408–14. doi: 10.1021/acs.inorgchem.4c05123 (PMC11898167; doi:10.1021/acs.inorgchem.4c05123)
Supplement: Supplementary file 1 — ic4c05123_si_001.pdf [file ic4c05123_si_001.pdf]

Supporting Information

Unique Organic-Inorganic Hybrid Copper(I) Phosphate with Ultralow Ractopamine Detection Limit and In-Situ Sensing Ability

Ji-Fang Xie,[a,+] Pi-Chen Wei,[a,+] Ying Li,[a,+] Chiao-Chun Chang,[a] Kai-Chi Chang,[b] Ching-Ping Lu,[c] Todd Hsu,[a] Der-Lii M Tzou,[d] Hsiung-Lin Tu,*[,d] and Chih-Min Wang*[,a,e]

[a]Department of Bioscience and Biotechnology, National Taiwan Ocean University, Keelung, Taiwan 202, R.O.C.

[b]Bachelor Degree Program in Marine Biotechnology, National Taiwan Ocean University, Keelung, Taiwan 202, R.O.C.

[c]Department of Environmental Biology and Fisheries Science, National Taiwan Ocean University, Keelung, Taiwan 202, R.O.C.

[d]General Education Center, National Taiwan Ocean University, Keelung, Taiwan 202, R.O.C.

[e]Institute of Chemistry, Academia Sinica, Taipei 11529, Taiwan.

[+]These authors contributed equally to this work.

Corresponding Author

*E-mail: twcmwang@gmail.com or cmwang@ntou.edu.tw (C.M.Wang).

*E-mail: hltu@gate.sinica.edu.tw (H. L. Tu).

1. Experimental Section

1.1 Synthesis of **NTOU-10**

The pure crystalline phase of **NTOU-10** was synthesized by heating a reaction mixture consisting of $Cu(NO_3)_2 \cdot 3H_2O$ (0.25 mmol), 3,5-dipyridyl-1,2,4-triazole (0.25 mmol), H_3PO_4 (0.25 mmol), HF (aqueous) (1 mmol, 48% solution), H_2O (1 mL), and 1,4-butanediol (3 mL) in a 20 mL Teflon-lined autoclave at 150°C for 2 days. The resulting product was filtered, washed with water and ethanol, dried in an oven, and used in subsequent experiments.

1.2 Characterizations

Single-crystal X-ray diffraction (SCXD)

SCXD intensity data were collected using a Rigaku XtaLAB Synergy DW diffractometer. All data calculations were performed using the PC versions of SHELXTL and Olex2 program packages. The crystal structure was visualized using Diamond 3.0.

Powder X-ray diffraction (PXRD)

Room-temperature PXRD patterns were recorded on a Bruker D2 PHASER desktop diffractometer with 300 W power (40 kV, 40 mA) using Cu Kα radiation (λ = 1.5418 Å) to determine the crystal structure.

Fourier transform infrared (FT-IR)

FT-IR spectra were obtained by a PerkinElmer spectrum 100 infrared spectrometer in the scan range of 4000~400 cm^{-1}.

X-ray photoelectron spectra (XPS)

XPS measurements were performed using a ULVAC PHI Quantera II with a scanning X-ray microprobe (Al anode).

Thermogravimetric analysis (TGA)

TGA curves were measured using a Perkin-Elmer TGA7 thermal analyzer. Samples were held in platinum pans and heated at a constant rate of $5°C \cdot min^{-1}$ from 45°C to 900°C under a continuous O_2 flow.

Elemental analysis (EA)

Elemental analysis was performed using a Foss Heraeus CHN-O-Rapid analyzer (Thermo FLASH EA 1112 Series) with 5 mg of each sample.

Nuclear magnetic resonance (NMR) spectroscopy

The ^{31}P cross polarization (CP) MAS NMR spectrum was recorded on a Bruker Advance 300 MHz spectrometer.

Dynamic light scattering analyzer (DLS)

Particle size distribution was measured using a Malvern DLS analyzer with an automatic attenuator. The intensity distribution and peak diameter were determined with a 633 nm He–Ne laser from the autocorrelation function under the general mode.

Scanning electron microscopy (SEM) and Energy-dispersive X-ray spectroscopy (EDX)

SEM images were obtained using a Zeiss Ultra Plus microscope with an accelerating voltage of 10 to 15 kV and a working distance of 10.0 mm. Samples were washed with water and ethanol, then dispersed onto carbon tape for mounting before SEM-EDX analysis.

1.3 Electrochemical measurement

All electrochemical experiments were conducted using the model 4054c, 621b, or 7211e electrochemical workstation (CH Instruments, Austin, TX). Dissolved oxygen in the electrolyte was removed by purging with high-purity nitrogen gas. The experiments were performed using a standard three-electrode system at room temperature.

Pretreatment of Reference Electrode

The screen-printed carbon electrode (SPCE; Zensor R&D, Taiwan) was pretreated by first dropping aqueous hydrogen peroxide (H_2O_2; >30%) and potassium chloride (KCl; saturated) on the electrode surface, followed by careful washing with deionized (DI) water. The bare electrode was then immersed in sulfuric acid (H_2SO_4; 0.1 M) and subjected to cyclic voltammetry (CV) to remove surface oxides. This was done by scanning the electrode for 10 cycles from –1.2 V to 1.2 V.

Preparation of **NTOU-10** Modified Electrode

Crystalline **NTOU-10** was continuously stirred in 0.1 M phosphate-buffered (PB) solution at pH 7.0 for 4 hours, yielding a fine powder. The modified electrode was prepared by depositing this fine powder onto either the SPCE or custom multi-electrode arrays. Optimal protocols were followed to develop electrodes for electrochemical detection of ractopamine (RAC) in 0.1 M PB solution at pH 7.0.

Formation of Hybrid Materials

To identify optimal hybrid materials that enhance electrochemical properties, three materials, polyglycolic acid (PGA), gold nanoparticles (Au NPs), and polypyrrole (PPy), were synthesized with **NTOU-10** powder. This was achieved through either the layer-by-layer (LBL) method or co-polymerization/deposition techniques, as illustrated in Figure S7. The detection performance of 0.1 μM RAC using differential pulse voltammetry (DPV) with these hybrid materials is shown in Figure S8. Among the hybrids, **NTOU-10@PPy** exhibited the highest sensitivity toward RAC and was therefore selected for this study.

Sensing Performance

Calibration experiments were conducted using DPV or amperometry (i-t curve) at an applied potential of 0.67 V in the electrolyte containing RAC. The current signal was calculated using the following equation:

$$\text{Normalized signal} = \Delta I / I_0 \ (1)$$

Where I is the electrochemical current in the presence of the RCA, I_0 is the background current in the absence of the RCA and $\Delta I = I - I_0$.

To determine the optimal synthetic protocol for **NTOU-10@PPy** as an ultrasensitive RAC sensor, we systematically examined several parameters: the concentration of **NTOU-10** and pyrrole monomer, as well as the scan rate and number of cycles during electrochemical polymerization, as shown in Figure S9. The modified electrode was prepared by cyclic voltammetry (CV) in a 0.1 M phosphate buffer (PB) solution (pH 7.0) containing a mixture of **NTOU-10** and pyrrole monomer, with a scan range from –0.25 to 0.75 V. The optimal concentrations for **NTOU-10** and pyrrole were determined to be 1.5 mg·mL^{-1} and 0.1 M, respectively. The highest current response was achieved at a scan rate of 100 mV·s^{-1} with 5 scan cycles. After synthesis, the electrodes were thoroughly washed with deionized (DI) water before further electrochemical measurements. The electrochemical quartz crystal microbalance (EQCM) technique was employed to detect frequency changes of a vibrating quartz resonator during surface processes in liquid electrolytes. The quartz resonance frequency shift (Δf) was converted into mass change (Δm) on the Au-coated quartz during the optimal CV-based synthetic protocol, using Sauerbrey's equation (2). The calibration constant applied in this work was 1.4 ng/Hz. The mass of **NTOU-10@PPy** deposited on the electrode surface during polymerization was calculated to be 173.8 ng, as derived from CV data (Figure S12). The electrode surface area was 0.07 cm²

$$\Delta f = \frac{2f_0^2}{A\sqrt{\rho_q \mu_q}} \Delta m \ (2)$$

where f_0 is the resonant frequency of the fundamental mode (Hz), Δf is the normalized frequency change (Hz), Δm is the mass change (g), A is the piezoelectrically active crystal area (1.47 cm^2), ρ_q is the density of quartz (2.648 g/cm^3), μ_q is the shear modulus of quartz for AT-cut crystal (2.947 × 10^{11} $g \cdot cm^{-1} \cdot s^{-2}$). Additionally, the kinetics of electrode reactions were investigated using cyclic voltammetry (CV) and electrochemical impedance spectroscopy (EIS; AC mode) in a 0.1 M KCl solution containing 5 mM ferri-ferrocyanide [$Fe(CN)_6^{3-/4-}$]. The impedance spectra were displayed as Nyquist plots for various modified electrodes. All modified electrodes showed semicircles with different diameters, corresponding to the charge transfer resistance, over the frequency range of 0.1 to 100 kHz

1.4 Practicability testing

Preparation of real samples

Fresh meat samples were purchased from a local supermarket and pretreated following the reported methods.[S9,S11] First, 2.0 g of minced meat was mixed with 4 mL of 0.1 $mol \cdot L^{-1}$ $HClO_4$ and homogenized for 20 minutes in an ultrasonic bath. The clear liquid phase was collected after heating the mixture at 80°C for 30 minutes, followed by centrifugation at 10,000 rpm for 10 minutes. The pH of the liquid solution was adjusted to 10 using Na_2CO_3 and NaCl. A 2 mL solution of 0.1 $mol \cdot L^{-1}$ HCl containing RAC was diluted with 0.1 $mol \cdot L^{-1}$ phosphate buffer (PB, pH 7.0). Samples with varying RAC concentrations (1 to 100 × 10^{-9} $mol \cdot L^{-1}$) were prepared for analysis (Table S3).

Real-time sensing of RAC using multi-electrode arrays

The substrate, a glass slide (7.5 × 2.5 cm^2), was cleaned by sequential sonication in aqueous Micro-90® (2% solution), isopropanol (IPA; 95%), and deionized water for 10 minutes. A metal mask for the multi-electrode arrays was designed using AutoCAD software (Autodesk, CA, USA) and fabricated by ANSY Electronics Co., Ltd. (Taiwan). The custom multi-electrode arrays were produced via sputter deposition of an Au target at a current of 30 mA, for 500 seconds, with a rotation speed of 100 rpm (Figure S16a). The multi-electrode arrays were integrated with an electrochemical flow injection analysis (FIA) system, where reagent flow was controlled by a syringe pump (KDS100, KD Scientific). This integrated system required less analyte compared to conventional methods, allowing for rapid detection and shorter reaction times (< 5 seconds) (Figure S16).

2. Figures

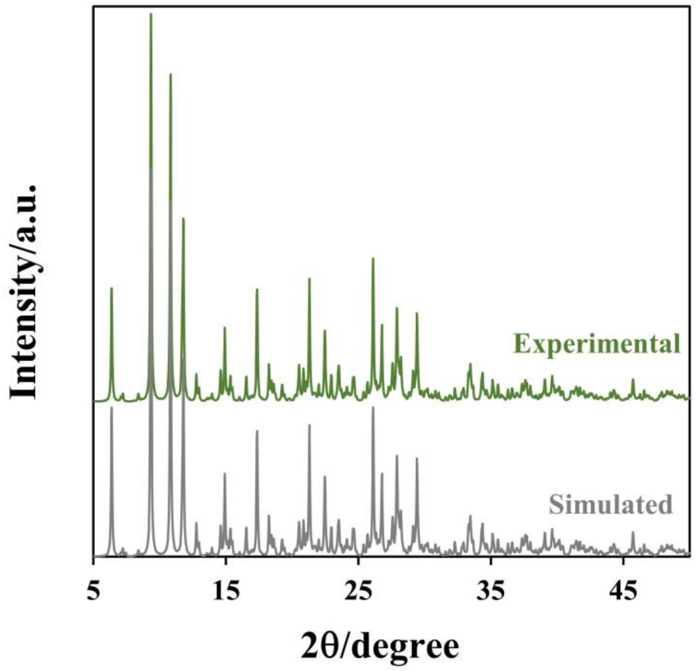

Figure S1. PXRD patterns for **NTOU-10**. Experimental and simulated patterns are shown in green and grey, respectively.

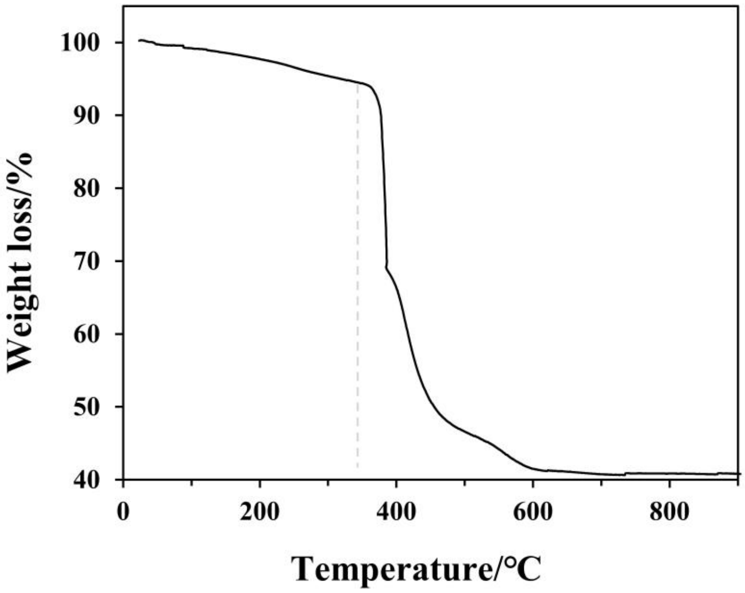

Figure S2. TGA curve in flowing O_2 gas for **NTOU-10**.

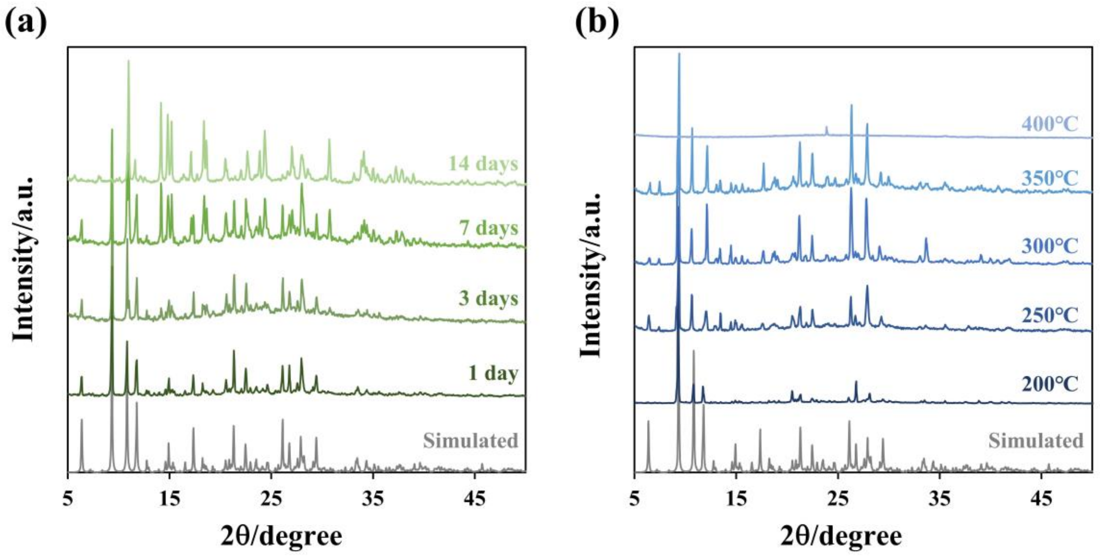

Figure S3. PXRD patterns of **NTOU-10** for stability studies at variable (a) time and (b) temperature.

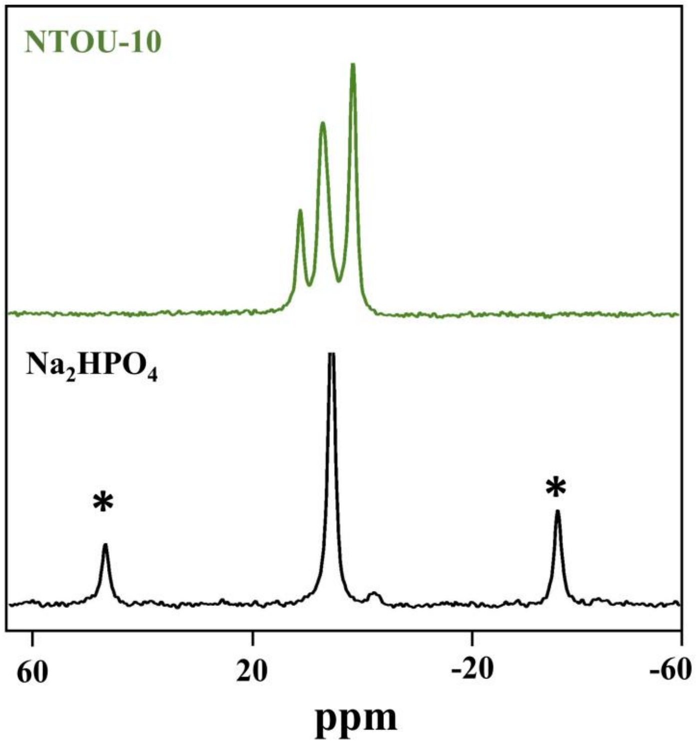

Figure S4. Solid-state ^{31}P CP/MAS NMR spectra analysis. Spinning sidebands are labeled with asterisks.

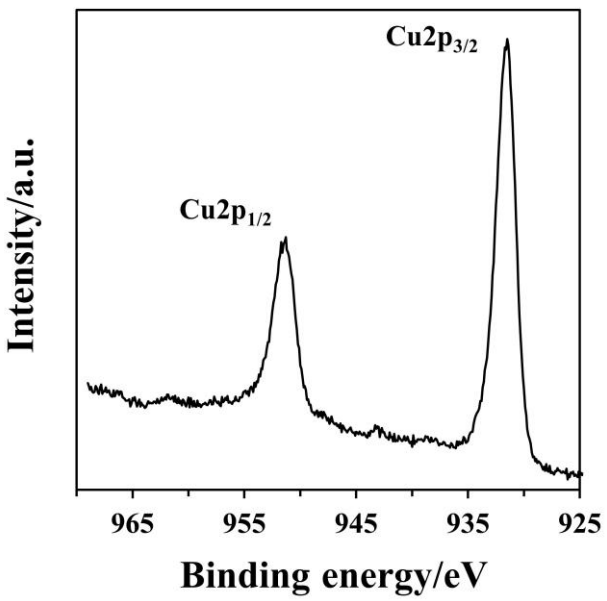

Figure S5. XPS analysis for Cu 2p spectral lines of **NTOU-10**.

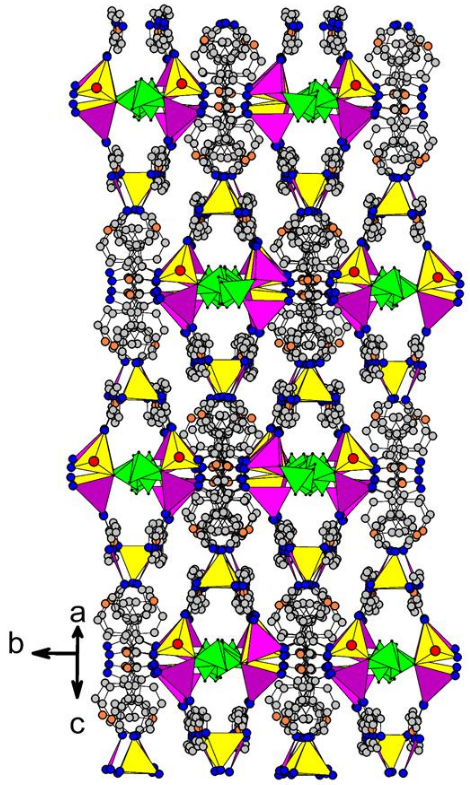

Figure S6. An organic-inorganic hybrid framework of **NTOU-10**. Blue, grey, and red circles represented N, C, and O atoms, respectively. The coordinated water oxygen atoms and nitrogen atoms of organic molecules not bonding to metals are separately in orange and large red circles. The phosphorous and copper polyhedra are indicated in green and yellow (or pink), respectively. The disordered lattice water molecules and hydrogen atoms are omitted for clarity.

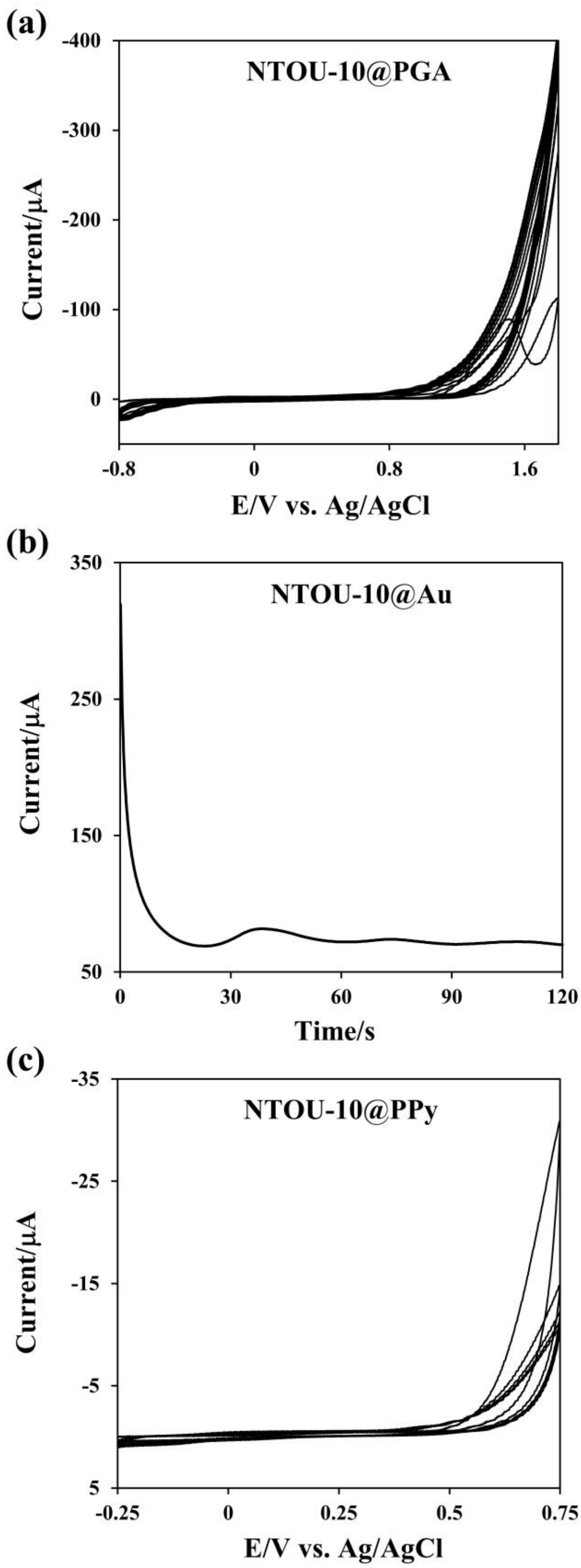

Figure S7. Cyclic voltammetry (CVs) and amperometry recorded during the polymerization/deposition of the hybrids modified electrodes of (a) **NTOU-10**@PGA, (b) **NTOU-10**@Au, and (c) **NTOU-10@PPy**.

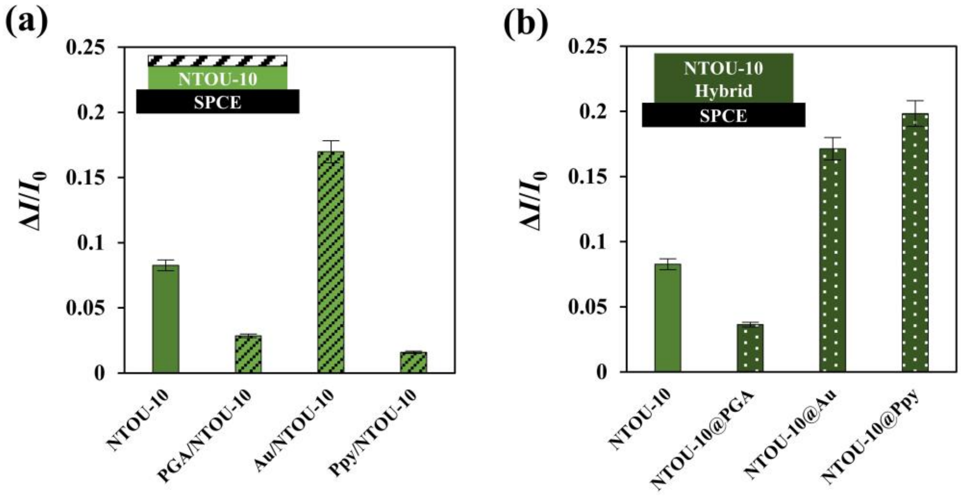

Figure S8. Differential pulse voltammetry (DPV) responses of various **NTOU-10** hybrids modified electrodes at (a) layer by layer and (b) co-polymerization/deposition. The experiments were performed in the 0.1 M pH 7.0 PB solution containing 10^{-7} mol·L^{-1} RAC.

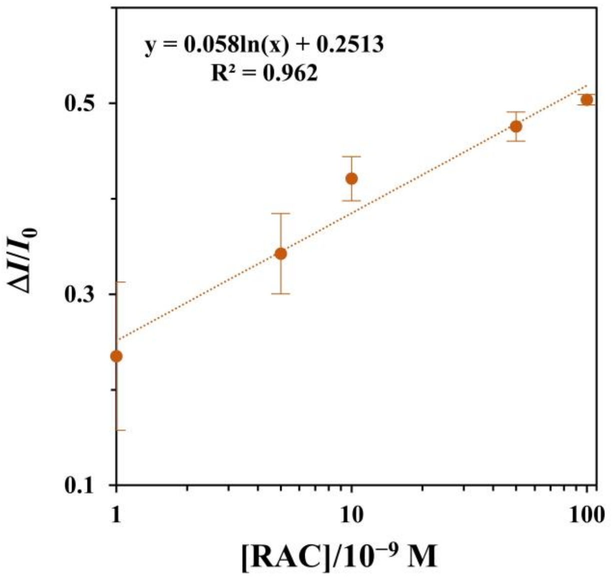

Figure S9. Calibration curve showed the current responses of **NTOU-10@PPy** in the presence of RAC concentrations ranging from 10^{-9} to 10^{-7} mol·L^{-1}.

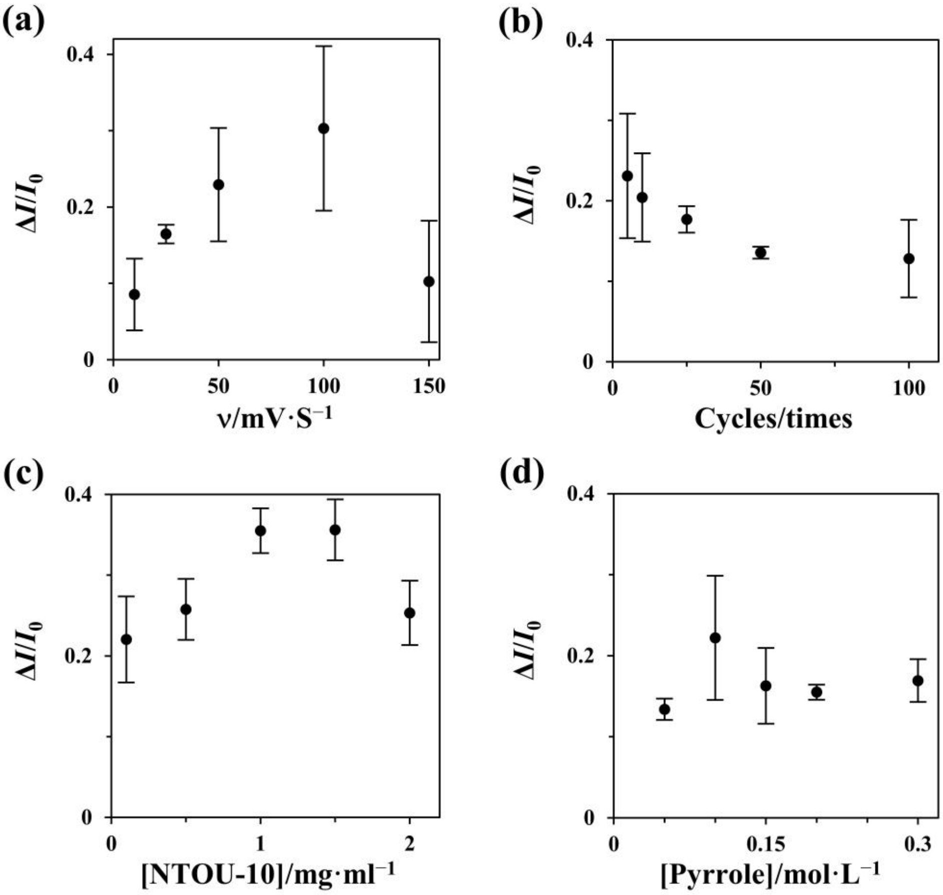

Figure S10. Optimization of the **NTOU-10@PPy** sensing performance. Amperometry (*i-t* curve) was used to determine the optimal electrochemical responses for RAC sensing (10^{-18} mol·L^{-1}) prepared using various experimental parameters, including the (a) scan rate and (b) running cycles, as well as the concentration of (c) **NTOU-10** and (d) pyrrole during electrical polymerization of the **NTOU-10@PPy** in the 0.1 M pH 7.0 PB solution.

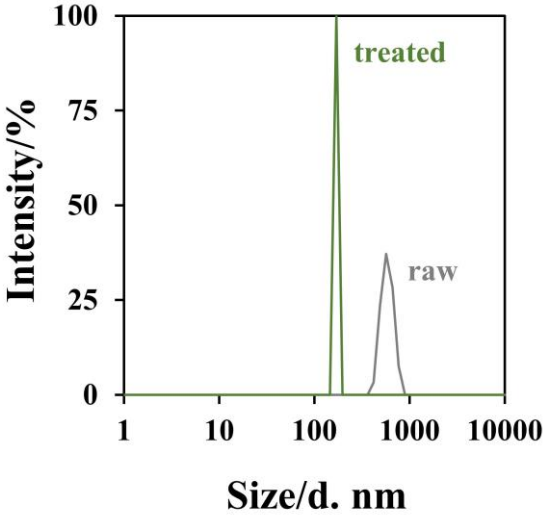

Figure S11. DLS analysis of particle size distribution for raw and treated samples.

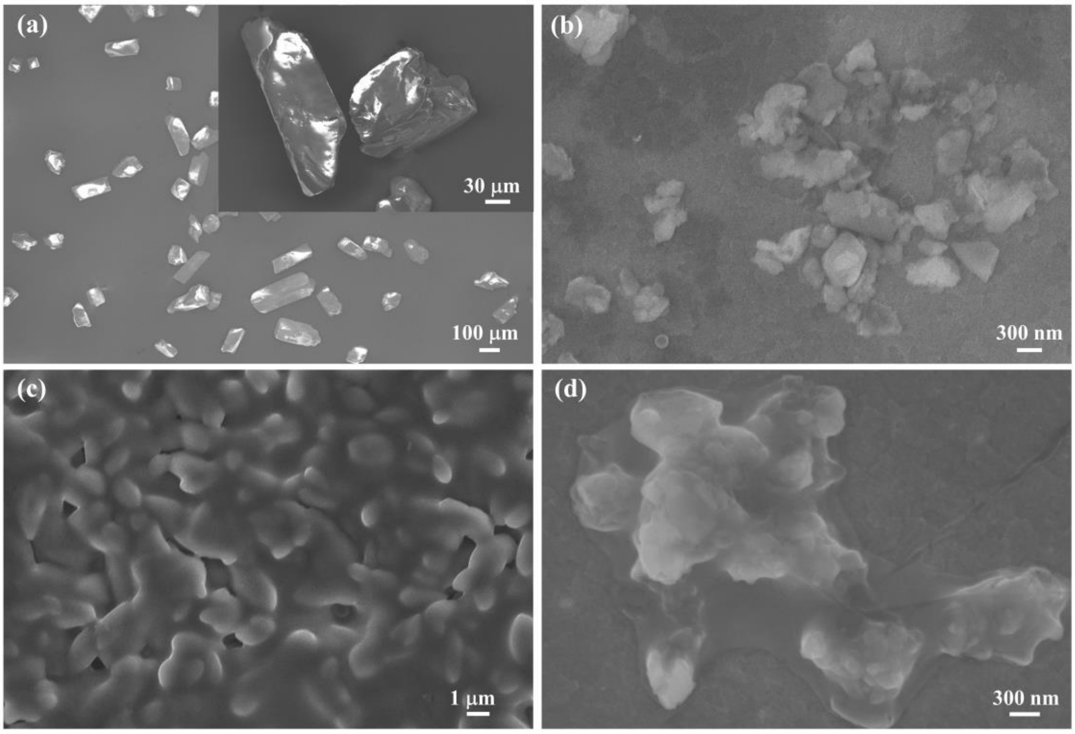

Figure S12. SEM images for (a) raw and (b) treated **NTOU-10**, as well as (c) PPy, and (d) **NTOU-10@PPy**.

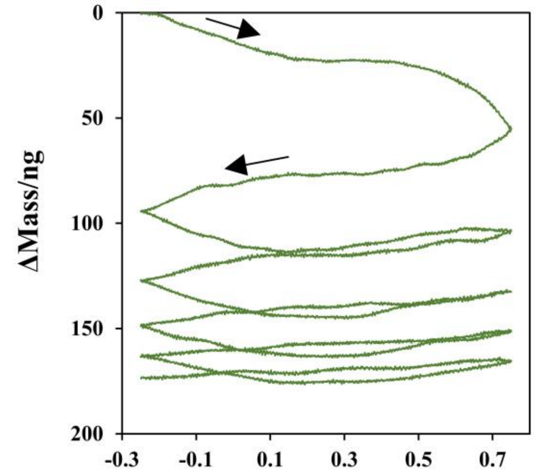

Figure S13. The EQCM responses for mass change (Δm) vs potential during the polymerization of **NTOU-10@PPy**.

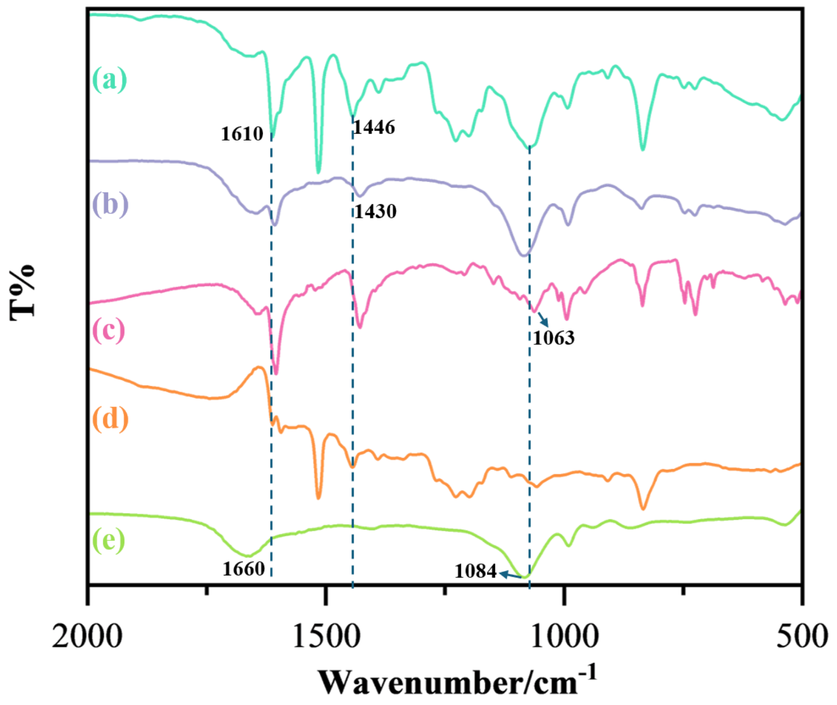

Figure S14. FTIR spectra of **NTOU-10@PPy-RAC** (a), **NTOU-10@PPy** (b). **NTOU-10** (c), RAC (d), and PPy (e).

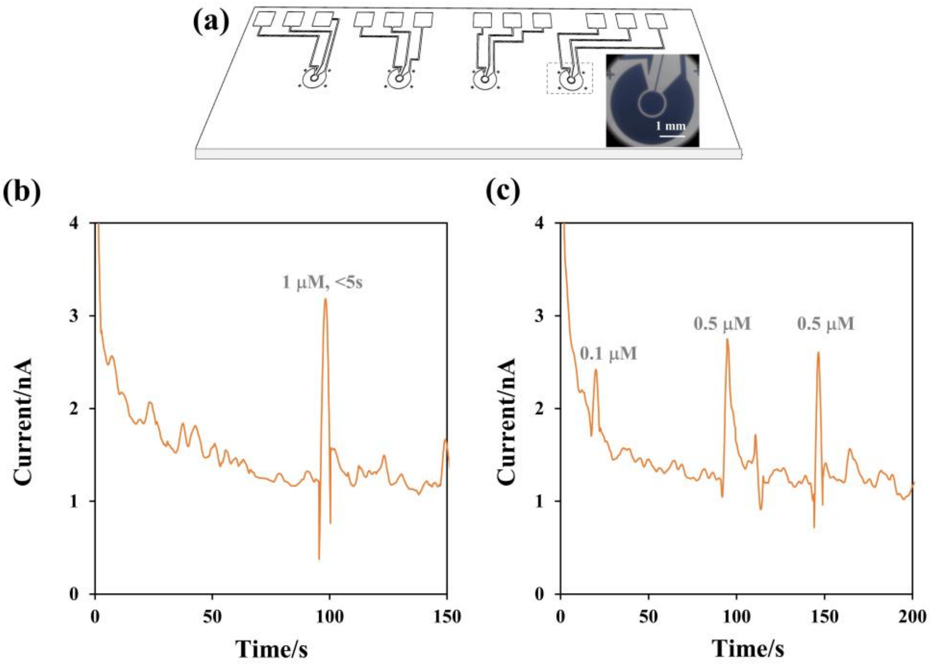

Figure S15. Integration of multi-electrode arrays with flow injection analysis: (a) The design and zoom-in photograph of multi electrode arrays. (b) and (c) The representative trace of flow injection analysis for ractopamine.

Table S1. Crystallographic Data for $Cu_9(H_2O)_2(HTDP)(TDP)_5(H_2PO_4)_{0.5}(H_{1.5}PO_4)(HPO_4)\cdot 2H_2O$ (**NTOU-10**).

**NTOU-10**	
Crystal size, mm	0.15 × 0.03 × 0.02
Crystal system	monoclinic
Space group	*I*-2/*a* (No. 1502)
a, Å	28.0251(3)
b, Å	12.9426(2)
c, Å	41.2172(4)
α, deg	90
β, deg	97.607(1)
γ, deg	90
V, Å^3	14818.6(3)
Z	4
fw	4351.463
T, K	100(1)
λ(Cu Kα), Å	1.54184
ρ_{calc}, g·cm^{-3}	1.950
μ(Cu Kα), mm^{-1}	4.010
θ_{max}, deg	77.19
Unique data (I > 2σ(I))	12791
$R_1{}^a$	0.0414
$wR_2{}^b$	0.1138

$^a R_1 = \Sigma||F_O| - |F_C||/\Sigma|F_O|$.

$^b wR_2 = \Sigma \{[w(F_O{}^2 - F_C{}^2)^2]/\Sigma[w(F_O{}^2)^2]\}^{1/2}$, $w = 1/[\sigma^2(F_o{}^2) + (aP)^2 + bP]$, $P = [Max(F_o,0) + 2(F_c)^2]/3$, where $a = 0.0580$ and $b = 47.4212$.

Table S2. Selected bond lengths (Å) for **NTOU-10**.

Cu(1)-N(1)	2.008(2)	Cu(1)-N(17)	2.353(3)
Cu(1)-N(11)	2.044(2)	Cu(1)-N(21)	2.041(2)
Cu(2)-O(1)	2.204(2)	Cu(2)-N(8)	1.927(3)
Cu(2)-N(4)	1.938(2)		
Cu(3)-O(5)	2.061(2)	Cu(3)-N(13)	1.954(3)
Cu(3)-N(3)	1.922(2)		
Cu(4)-O(3)	2.131(2)	Cu(4)-N(19)	1.951(2)
Cu(4)-N(9)	1.928(3)		
Cu(5)-O(5)	2.200(2)	Cu(5)-N(18)	1.941(3)
Cu(5)-N(14)	1.940(3)	Cu(5)-OW1	2.424(4)
Cu(6)-N(5)	1.997(2)	Cu(6)-N(30)	2.026(3)
Cu(6)-N(20)	1.979(2)		
Cu(7)-N(10)	2.044(2)	Cu(7)-N(25)	1.954(2)
Cu(7)-N(16)	1.961(2)		
Cu(8)-O(9)	2.149(2)	Cu(8)-N(29)	1.913(2)
Cu(8)-N(24)	1.914(2)		
Cu(9)-O(9)	2.354(2)	Cu(9)-N(28)	1.940(3)
Cu(9)-N(23)	1.946(2)	Cu(9)-OW2	2.400(3)
P(1)-O(1)	1.544(3)	P(1)-O(3)	1.514(2)
P(1)-O(2)	1.532(2)	P(1)-O(4)	1.591(3)
P(2)-O(5)	1.503(3)	P(2)-O(7)	1.557(2)
P(2)-O(6)	1.575(2)	P(2)-O(8)	1.524(3)
P(3)-O(9) (2 x)	1.541(2)	P(3)-O(10) (2 x)	1.548(2)

Table S3. Selected Bond Angle (deg) for **NTOU-10**.

N(11)-Cu(1)-N(1)	114.1(1)	N(21)-Cu(1)-N(1)	120.0(1)
N(17)-Cu(1)-N(1)	104.8(1)	N(21)-Cu(1)-N(11)	110.9(1)
N(17)-Cu(1)-N(11)	99.6(1)	N(21)-Cu(1)-N(17)	104.6(9)
N(4)-Cu(2)-O(1)	109.2(1)	N(8)-Cu(2)-N(4)	147.3(1)
N(8)-Cu(2)-O(1)	103.4(1)		
N(3)-Cu(3)-O(5)	121.2(1)	N(13)-Cu(3)-N(3)	139.5(1)
N(13)-Cu(3)-O(5)	98.7(1)		
N(9)-Cu(4)-O(3)	116.9(1)	N(13)-Cu(4)-N(9)	138.7(1)
N(19)-Cu(4)-O(3)	104.3(1)		
N(14)-Cu(5)-O(5)	97.19(9)	OW1-Cu(5)-O(5)	88.1(1)
N(18)-Cu(5)-O(5)	104.3(1)	OW1-Cu(5)-N(14)	89.0(1)
N(18)-Cu(5)-N(14)	148.5(1)	OW1-Cu(5)-N(18)	114.1(1)
N(20)-Cu(6)-N(5)	124.3(1)	N(30)-Cu(6)-N(20)	111.8(1)
N(30)-Cu(6)-N(5)	114.4(1)		
N(16)-Cu(7)-N(10)	109.9(1)	N(25)-Cu(7)-N(16)	135.4(1)
N(25)-Cu(7)-N(10)	113.9(1)		
N(24)-Cu(8)-O(9)	109.87(9)	N(29)-Cu(8)-N(24)	146.2(1)
N(29)-Cu(8)-O(9)	103.20(9)		
N(23)-Cu(9)-O(9)	104.12(9)	OW2-Cu(9)-O(9)	91.67(9)
N(28)-Cu(9)-O(9)	98.05(9)	OW2-Cu(9)-N(23)	118.8(1)
N(28)-Cu(9)-N(23)	145.9(1)	OW2-Cu(9)-N(28)	85.7(1)
O(2)-P(1)-O(1)	114.0(1)	O(4)-P(1)-O(1)	108.5(1)
O(3)-P(1)-O(1)	109.7(1)	O(4)-P(1)-O(2)	108.3(1)
O(3)-P(1)-O(2)	112.6(1)	O(4)-P(1)-O(3)	106.5(1)
O(6)-P(2)-O(5)	114.0(1)	O(8)-P(2)-O(5)	108.5(1)
O(7)-P(2)-O(5)	109.7(1)	O(8)-P(2)-O(6)	108.3(1)
O(7)-P(2)-O(6)	112.6(1)	O(8)-P(2)-O(7)	106.5(1)
O(9)-P(3)-O(9)	111.7(2)	O(10)-P(3)-O(9) (2 x)	108.7(1)
O(9)-P(3)-O(10) (2 x)	109.7(1)	O(10)-P(3)-O(10)	109.3(2)

The P(3) atom was located in the special position.

Table S4. Comparison of different sensors for detection of RAC

No	Sensors	Technique	Detection limit ($mol·L^{-1}$)	Linear range ($mol·L^{-1}$)	References
1	GO/GCE	DPV	5.64×10^{-8}	8.29×10^{-8}— 3.32×10^{-5}	S1
2	MIP/gold electrode		2.66×10^{-8}	2.24×10^{-7}—1.57×10^{-6}	S2
3	MWCNT/MIM/SPE		6.74×10^{-9}	2.24×10^{-8}—2.24×10^{-7}	S3
4	MIP/AuNPs–GR/GCE		1.53×10^{-9}	3.32×10^{-8}—1.66×10^{-5}	S4
5	Aptamer/AuNPs/PDDA-GN/GCE	DPV	5.0×10^{-13}	1.0×10^{-12}— 1.0×10^{-8}	S5
6	C_3N_4NTs/ILs/CPE	DPV	1.0×10^{-13}	1.0×10^{-9}—1.0×10^{-12}	S6
7	dsDNA/CuNPs		3.0×10^{-13}	1.0×10^{-12}—3.0×10^{-7}	S7
8	Aptamer/AuNPs/JPs/GCE		3.3×10^{-14}	1.0×10^{-13}—1.53×10^{-9}	S8
9	AB/GCE		1.66×10^{-9}	3.33×10^{-9}— 6.66×10^{-6}	S9
10	$PMEO_2MA/C_{60}$-rGO/GCE		8.2×10^{-8}	1.0×10^{-7}— 3.1×10^{-6}	S10
11	Fe_3O_4/rGO/MSPE	DPV	1.3×10^{-8}	5.0×10^{-8}—1.0×10^{-5}	S11
12	NTOU-10@PPy	i-t	3.31×10^{-19}	1.0×10^{-18}—1.0×10^{-16}	This work

GO: graphene oxide, **GCE**: glassy carbon electrode, **MIP/M**: molecularly imprinted polymer/membranes, **GR**: graphene, **PDDA-GN**: poly dimethyl diallyl ammonium chloride–graphene, **Ils**: ionic liquid, **CPE**: carbon paste electrode, **JPs**: octadecanethiol Janus particles, **AB**: acetylene black, **PMEO₂MA**: poly(2-(2-methoxyethoxy)ethyl methacrylate), **rGO**: reduced graphene oxide, **MSPE**: magnetic screen-printed electrode

Table S5. Practical performance test using pork meat with spiked-in RAC

Spiked (10^{-9} mol·L^{-1})	Detected (10^{-9} mol·L^{-1})	Recovery (%)
1	1.02	102
5	4.89	97.8
10	10.82	108.2
50	50.13	100.3
100	98.24	98.24

References

[S1] C. Wu, D. Sun, Q. Li, K. B. Wu. Electrochemical sensor for toxic ractopamine and clenbuterol based on the enhancement effect of graphene oxide. *Sens. actuators. B Chem.* **2012**, *168*, 178-184.

[S2] L. J. Kong, M. F. Pan, G. Z. Fang, K. Qian, S. Wang. An electrochemical sensor for rapid determination of ractopamine based on a molecularly imprinted electrosynthesized o-aminothiophenol film. *Anal Bioanal Chem.* **2012**, *404*, 1653-1660.

[S3] H. C. Zhang, G. Y. Liu, C. Y. Chai. A novel amperometric sensor based on screen-printed electrode modified with multi-walled carbon nanotubes and molecularly imprinted membrane for rapid determination of ractopamine in pig urine. *Sens. actuators. B Chem.* **2012**, *168*, 103-110.

[S4] T. F. Li, T. Yao, C. Zhang, G. Y. Liu, Y. X. She, M. J. Jin, F. Jin, S. S. Wang, H. Shao, J. Wang. Electrochemical detection of ractopamine based on a molecularly imprinted poly-o-phenylenediamine/gold nanoparticle–ionic liquid–graphene film modified glass carbon electrode. *Rsc Adv.* **2016**, *6*, 66949-66956.

[S5] F. Yang, P. L. Wang, R. G. Wang, Y. Zhou, X. O. Su, Y. J. He, L. Shi, D. S. Yao. Label free electrochemical aptasensor for ultrasensitive detection of ractopamine. *Biosens Bioelectron.* **2016**, *77*, 347-352.

[S6] S. Mert, B. Bankoglu, A. Özkan, N. Atar, M. L. Yola. Electrochemical sensing of ractopamine by carbon nitride nanotubes/ionic liquid nanohybrid in presence of other β-agonists. *J. Mol. Liq.* **2018**, *254*, 8-11.

[S7] F. F. Sheng, X. J. Zhang, G. F. Wang. Novel ultrasensitive homogeneous electrochemical aptasensor based on dsDNA-templated copper nanoparticles for the detection of ractopamine. *J. Mater. Chem. B.* **2017**, *5*, 53-61.

[S8] Y. Zhou, Y. J. Yang, X. Deng, G. M. Zhang, Y. Zhang, C. H. Zhang, S. M. Shuang, Y. J. He, W. Sun. Electrochemical sensor for determination of ractopamine based on aptamer/octadecanethiol Janus particles. *Sens. actuators. B Chem.* **2018**, *276*, 204-210.

[S9] R. Wang, K. B. Wu, C. Wu. Highly sensitive electrochemical sensor for toxic ractopamine based on the enhancement effect of acetylene black nanoparticles. *Anal. Methods.* **2015**, *7*, 8069-8077.

[S10] C. Chen, M. X. Zhang, C. Y. Li, Y. X. Xie, J. J. Fei. Switched voltammetric determination of ractopamine by using a temperature-responsive sensing film. *Microchimica Acta.* **2018**, *185*.

[S11] Y. Poo-arporn, S. Pakapongpan, N. Chanlek, R. P. Poo-arporn. The development of disposable electrochemical sensor based on Fe_3O_4-doped reduced graphene oxide modified magnetic screen-printed electrode for ractopamine determination in pork sample. *Sens. actuators. B Chem.* **2019**, *284*, 164-171.
